# Sodium alginate prevents progression of non-alcoholic steatohepatitis and liver carcinogenesis in obese and diabetic mice

**DOI:** 10.18632/oncotarget.7249

**Published:** 2016-02-08

**Authors:** Tsuneyuki Miyazaki, Yohei Shirakami, Masaya Kubota, Takayasu Ideta, Takahiro Kochi, Hiroyasu Sakai, Takuji Tanaka, Hisataka Moriwaki, Masahito Shimizu

**Affiliations:** ^1^ Department of Gastroenterology/Medicine, Gifu University Graduate School of Medicine, Gifu, Japan; ^2^ Informative Clinical Medicine, Gifu University Graduate School of Medicine, Gifu, Japan; ^3^ Tumor Pathology, Gifu University Graduate School of Medicine, Gifu, Japan

**Keywords:** obesity, non-alcoholic steatohepatitis, liver carcinogenesis, sodium alginate, diabetes mellitus

## Abstract

Obesity and related metabolic abnormalities play a key role in liver carcinogenesis. Non-alcoholic steatohepatitis (NASH), which is often complicated with obesity and diabetes mellitus, is associated with the development of hepatocellular carcinoma (HCC). Sodium alginate (SA), which is extracted from brown seaweeds, is marketed as a weight loss supplement because of its high viscosity and gelling properties. In the present study, we examined the effects of SA on the progression of NASH and related liver carcinogenesis in monosodium glutamate (MSG)-treated mice, which show obesity, diabetes mellitus, and NASH-like histopathological changes. Male MSG-mice were intraperitoneally injected with diethylnitrosamine at 2 weeks of age, and, thereafter, they received a basal diet containing high- or low-molecular-weight SA throughout the experiment (16 weeks). At sacrifice, control MSG-treated mice fed the basal-diet showed significant obesity, hyperinsulinemia, steatosis and hepatic tumor development. SA administration suppressed body weight gain; improved insulin sensitivity, hyperinsulinemia, and hyperleptinemia; attenuated inflammation in the liver and white adipose tissue; and inhibited hepatic lipogenesis and progression of NASH. SA also reduced oxidative stress and increased anti-oxidant enzyme levels in the liver. Development of hepatic tumors, including liver cell adenoma and HCC, and hepatic pre-neoplastic lesions was significantly inhibited by SA supplementation. In conclusion, oral SA supplementation improves liver steatosis, insulin resistance, chronic inflammation, and oxidative stress, preventing the development of liver tumorigenesis in obese and diabetic mice. SA may have ability to suppress steatosis-related liver carcinogenesis in obese and diabetic subjects.

## INTRODUCTION

Hepatocellular carcinoma (HCC) is a serious healthcare problem worldwide because of its increasing morbidity and high mortality. Chronic inflammation of the liver and subsequent cirrhosis are the strongest risk factors for HCC development. Recent evidence also indicates that obesity and related metabolic abnormalities, especially diabetes mellitus and insulin resistance, increase the risk of HCC [1–3]. Obesity promotes hepatic steatosis and inflammation through the production of pro-inflammatory cytokines such as tumor necrosis factor (TNF)-α and interleukin (IL)-6, which are closely associated with liver carcinogenesis [4, 5]. Increased levels of oxidative stress and aberrant lipogenesis in the liver, both of which are significantly linked to obesity and metabolic syndrome, are also dominantly observed during liver carcinogenesis and HCC progression [5, 6].

Non-alcoholic fatty liver disease (NAFLD) is a hepatic manifestation of the metabolic syndrome, and a proportion of patients with this disease can show progression to non-alcoholic steatohepatitis (NASH) and the risk of developing cirrhosis and HCC [7]. Amelioration of obesity, insulin resistance, and steatosis and reduction of oxidative stress are critical steps for inhibiting the development of NASH [8]. A recent clinical trial showed that treatment with vitamin E, an anti-oxidant, significantly improved hepatic histopathology in patients with NASH [9]. Therefore, in addition to lifestyle modification to reduce body weight, active pharmacotherapy is considered necessary for the management of patients with NASH, especially those who have the metabolic syndrome.

Recent preclinical and clinical studies have demonstrated that targeting obesity and related metabolic abnormalities, such as attenuation of chronic inflammation and improvement of insulin resistance, may be an effective strategy for preventing liver carcinogenesis in obese individuals[10, 11]. We have previously reported that supplementation with branched chain amino acids (BCAA), which prevents HCC development in cirrhotic patients with obesity [3], significantly inhibits liver carinogenesis in obese and diabetic mice by improving insulin resistance and attenuating chronic inflammation in both the liver and white adipose tissue (WAT) [12, 13].

Alginate is a gelling polysaccharide and a structural component extracted from marine brown algae. Alginate provides mechanical strength and fiexibility in seaweed, and the fiber is composed of mannuronic and guluronic acids, which influence its viscous physiological properties. For decades, the food industry has widely used alginates as additives because of their gelling, viscosifying, and stabilizing properties [14]. Ingestion of sodium alginate (SA), the most commonly used alginate, and subsequent gelation in the stomach appear to decrease the human appetite in acute settings [15]. An important rheological property of fibers within the intestine is viscosity, which is thought to account for the beneficial physiological responses in relation to appetite regulation [15], as well as glycemic and lipidemic control [16].

SA supplementation decreases body weight and improves obesity [17], which suggests that SA may be effective in the treatment of metabolic disorders as well as the overall symptoms of the metabolic syndrome caused by obesity. However, effects of SA on the inhibition of obesity- and NASH-related liver carcinogenesis have not yet been studied. The purpose of this study was to investigate whether SA supplementation would significantly inhibit hepatic carcinogen diethylnitrosamine (DEN)-induced liver tumorigenesis in monosodium glutamate (MSG)-treated mice, which is a model animal that shows obesity, hyperlipidemia, and diabetes mellitus and reflects steatosis-related liver carcinogenesis in human [18–20], by focusing on its anti-obesity, anti-diabetic, anti-inflammatory, and anti-oxidant effects. Moreover, since SA used in previous investigations is usually high-molecular-weight SA, we examine and compare the effects of low-molecular-weight SA, which is considered relatively easy to administer orally.

## RESULTS

### General observations

Body weights and relative weights of the liver of the mice that received high- and low-molecular-weight SA were significantly lower than those of SA-untreated control mice at the termination of the experiment, 21 weeks of age (Table 1; *P* < 0.05). Relative weights of WAT of SA-treated mice were higher than those of untreated mice (*P* < 0.05). During the experiment, SA showed no clinical symptoms of toxicity. Histopathological examinations also revealed the absence of toxicity due to SA in the liver, kidney, and spleen (data not shown). No significant difference was seen in the amount of food ingested by all groups during the experiment.

**Table 1:** Body, liver, and white adipose tissue weights of mice at the end of experiment, 21 weeks of age

Group no.	Treatment	No. of mice	Body weight (g)	Relative organ weight (g/100g body weight)	
				Liver	WAT^a^
G1	DEN	12	77.3 ± 6.8^b^	5.4 ± 1.2	3.3 ± 1.0
G2	DEN/High-molecular SA	12	60.6 ± 6.1^c^	4.2 ± 0.4^c^	5.1 ± 0.9^c^
G3	DEN/Low-molecular SA	12	61.2 ± 6.2^c^	4.1 ± 0.5^c^	5.1 ± 1.7^c^

a White adipose tissue of the periorchis and retroperitoneum.

b Mean ± SD.

c Significantly different from group 1 by Tukey-Kramer Multiple Comparison Test (*P* < 0.05).

### Effects of SA on DEN-induced liver tumorigenesis in MSG mice

**Figure 1:**
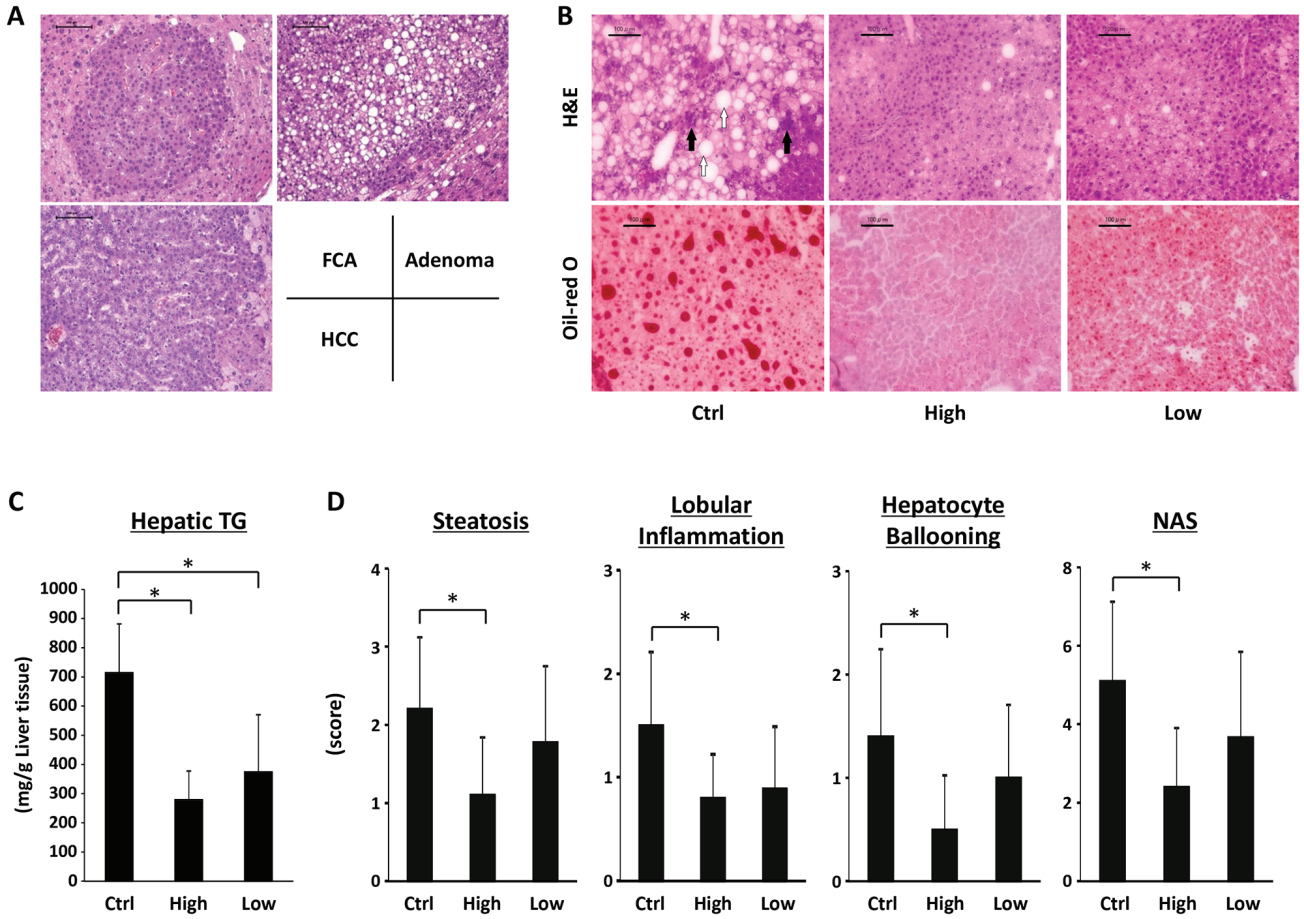
Effects of SA on the hepatic histopathology and intrahepatic triglyceride levels of the experimental mice **A.** Representative photomicrographs of foci of cellular alteration (FCA), liver cell adenoma and hepatocellular carcinoma (HCC). **B.** Representative photomicrographs of H&E staining (upper panels) and Oil-red O staining (lower panels) of liver sections from the control mice (left line), high-molecular SA-treated mice (middle line), and low-molecular SA-treated mice (right line) at the end of experiment, 21 weeks of age. Infiltration of inflammatory cells (indicated by black arrows) and ballooned hepatocytes (indicated by white arrows) were observed. Black bar: 100 μm. **C.** Hepatic lipids were extracted from frozen livers of the experimental mice, and triglyceride levels were measured. **D.** Presence of NAS (steatosis, inflammation, and ballooning) was determined using histopathological analysis. Values are expressed as mean ± SD. *P < 0.05. Ctrl; DEN-treated control group without SA administration. High; high-molecular SA-treated group. Low; low-molecular SA-treated group.

Nodular lesions (liver cell adenoma and HCC) and FCA (Figure 1A) were observed in the liver of the experimental mice in all groups at the end of the study. However, as listed in Table 2, treatment with low-molecular-weight SA significantly reduced the incidence of HCC and multiplicity of FCA and liver cell adenoma (*P* < 0.05). Incidence and multiplicity of adenoma were also significantly decreased in the liver of mice treated with high-molecular-weight SA (*P* < 0.05).

**Table 2:** Effects of sodium alginate on incidence and multiplicity of hepatic neoplasms and pre-neoplastic lesions in the experimental mice

Group no.	Treatment	No. of mice	Incidence			Multiplicity^a^		
			FCA^b^	Adenoma	HCC^c^	FCA	Adenoma	HCC
G1	DEN	12	10/10 (100%)	8/10 (80%)	5/10 (50%)	5.3 ± 3.4^d^	2.0 ± 2.2	1.1 ± 1.6
G2	DEN/High-molecular SA	12	10/10 (100%)	1/10^e^ (10%)	1/10 (10%)	3.0 ± 2.5	0.1 ± 0.3^f^	0.3 ± 0.9
G3	DEN/Low-molecular SA	12	8/9 (88.8%)	1/9 (11.1%)	0/9^e^ (0%)	1.4 ± 1.0^f^	0.1 ± 0.3^f^	0

a Number of neoplasms per mouse.

b FCA, foci of cellular alteration.

c HCC, hepatocellular carcinoma.

d Mean ± SD.

e Significantly different from group 1 by Fisher's exact probability test (*P* < 0.05).

f Significantly different from group 1 by Tukey-Kramer Multiple Comparison Test (*P* < 0.05).

### Effects of SA on hepatic histopathology and hepatic triglyceride levels in the experimental mice

Results of H&E and Oil-red O-staining of the livers of the experimental mice are presented in Figure 1B. Infiltration of inflammatory cells and ballooning degeneration of hepatocytes were observed in the MSG-treated mice. Examination of Oil-red O-stained sections revealed severe hepatic steatosis in SA-untreated control mice, but it was markedly improved by SA administration. Similar to the histological findings, intrahepatic TG levels were also significantly reduced by SA administration (Figure 1C, *P* < 0.05). Hepatic steatosis, lobular inflammation, ballooning degeneration of hepatocytes, and NAS were also significantly lower in the high-molecular-weight SA-supplemented mice than in the SA-untreated mice (Figure 1D, *P* < 0.05). No obvious liver fibrosis was seen in all groups.

### Effects of SA on expression levels of *FAS*, *SREBP1c*, *PPAR-α*, *TNF-α*, *IL-1β*, *F4/80*, and *CCL2* mRNAs in the liver of the experimental mice

**Figure 2:**
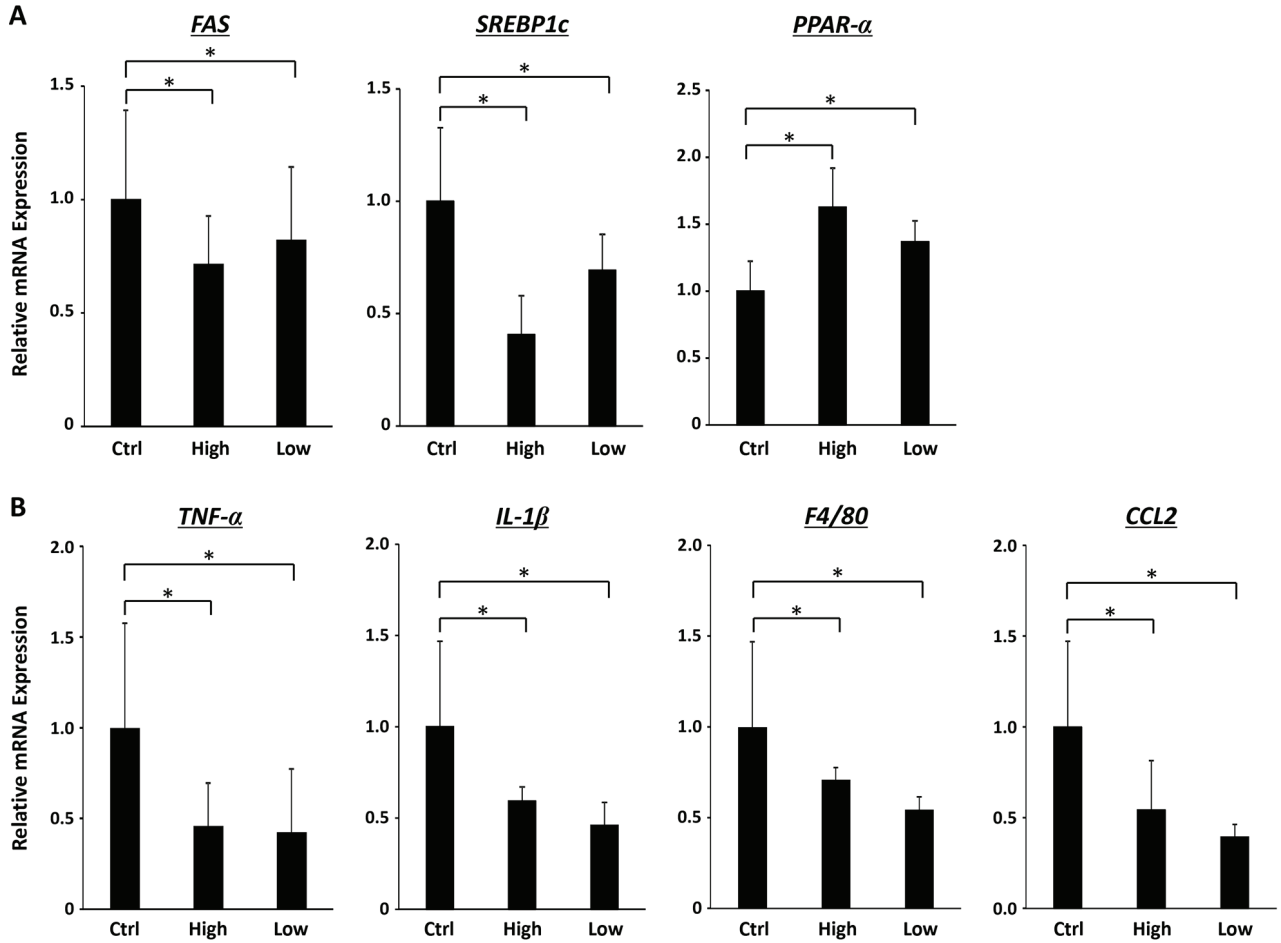
Effects of SA on the expression levels of mRNAs involved in lipid metabolism and inflammation in the liver of the experimental mice Total RNA was isolated from the livers of the experimental mice, and expression levels of mRNA associated with lipid metabolism (**A.** *FAS*, *SREBP1c* and *PPAR-α*) and inflammation (**B.** *TNF-α*, *IL-1β*, *F4/80* and *CCL2*) were determined using quantitative real-time RT-PCR with specific primers. Values are expressed as mean ± SD. *P < 0.05.

Because hepatic steatosis and lobular inflammation were improved by SA administration (Figure 1B, 1C, and 1D), effects of SA on expression levels of specific molecules that control hepatic lipid metabolism and inflammation were examined. High- and low-molecula-weight SA-treated mice showed significant decrease in expression levels of *FAS* and *SREBP1c* mRNAs (Figure 2A, *P* < 0.05), which are involved in fatty acid synthesis [21]. In contrast, mRNA expression levels of PPAR-α, an entry flux of fatty acid regulator [21], were increased by SA administration (*P* < 0.05). In addition, SA treatment significantly decreased mRNA expression levels of inflammatory mediators such as *TNF-α*, *IL-1β*, *F4/80*, and *CCL2*, in the liver of experimental mice (Figure 2B, *P* < 0.05).

### Effects of SA on systemic oxidative stress and hepatic expression of *Catalase* and *GPx1* mRNAs in the experimental mice

**Figure 3:**
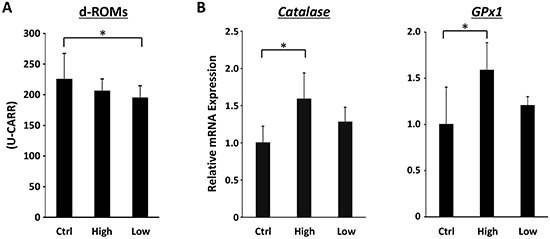
Effects of SA on oxidative stress in the experimental mice **A.** Hydroperoxide levels in the serum at the end of the experiment were determined using the d-ROM test. **B.** Total RNA was isolated from the livers of the experimental mice, and the expression levels of *Catalase* and *GPx1* mRNAs were examined using quantitative real-time RT-PCR with specific primers. Values are expressed as mean ± SD. *P < 0.05.

Hepatic oxidative stress is involved in the progression of fatty livers to NASH and subsequent HCC development [22, 23]. Therefore, levels of oxidative stress and antioxidant biomarkers in the experimental mice were assessed. Treatment with low-molecular-weight SA showed a significant decrease in serum d-ROM levels, which reflect serum hydroperoxide levels (Figure 3A, *P* < 0.05). In contrast, expression level of *Catalase* and *GPx1* mRNA, which encode antioxidant enzymes, were effectively increased by high-molecular-weight SA treatment (Figure 3B, *P* < 0.05).

### Effects of SA on expression levels of *TNF-α*, *IL-6*, *F4/80* and *CCL2* mRNAs in the adipose tissue of the experimental mice

**Figure 4:**
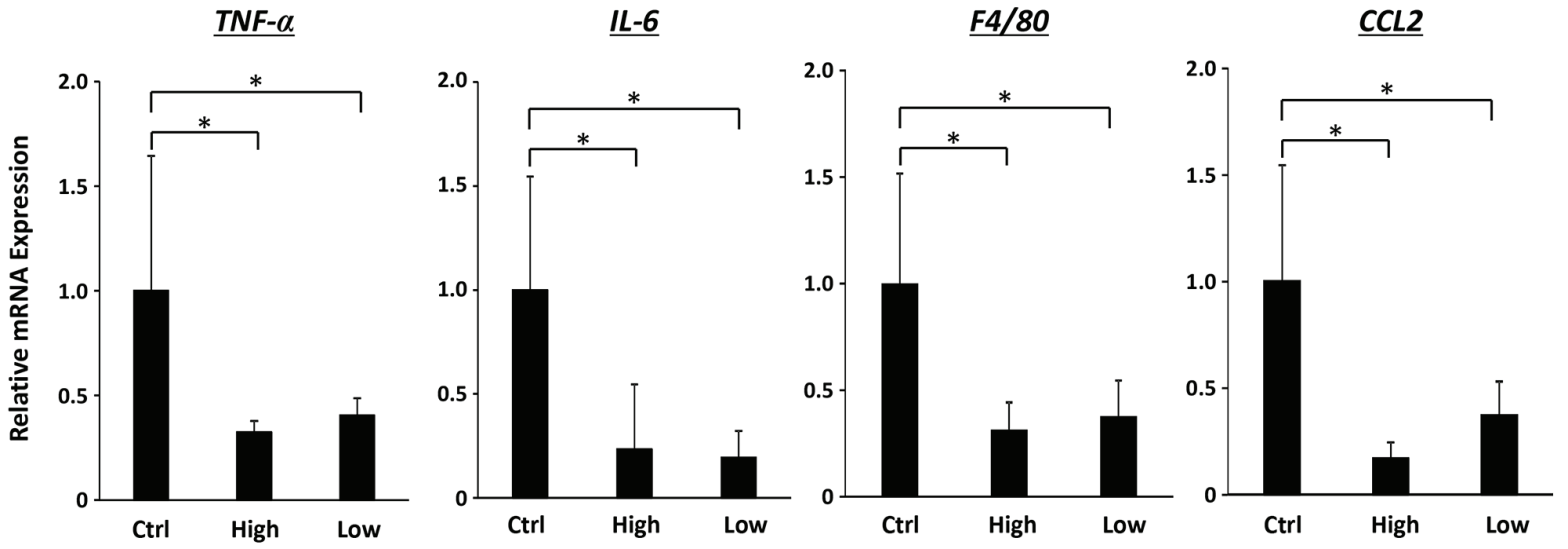
Effects of SA on expression levels of *TNF-α*, *IL-6*, *F4/80* and *CCL2* mRNAs in the WAT of the experimental mice Total RNA was isolated from the WAT of experimental mice, and expression levels of *TNF-α*, *IL-6*, *F4/80*, and *CCL2* mRNA were determined using quantitative real-time RT-PCR with specific primers. Values are expressed as mean ± SD. *P < 0.05.

Inflammation in the adipose tissue plays a key role in the pathophysiology of obesity [24]. Therefore, we checked whether SA treatment attenuates chronic inflammation in WAT. As shown in Figure 4, expression levels of *TNF-α* and *IL-6* mRNAs in the WAT were significantly reduced by SA treatment (*P* < 0.05). In addition, treatment with SA markedly inhibited mRNA expression of *F4/80* and *CCL2* (*P* < 0.05), which plays a role in the recruitment of macrophages into obese adipose tissue [24, 25].

### Effects of SA on serum parameters and insulin sensitivity in the experimental mice

Serum levels of AST and ALT were significantly decreased in SA-treated mice (Table 3, *P* < 0.05). SA treatment markedly decreased serum level of insulin and increased the value of QUICKI, which indicates that insulin resistance is improved by this agent (*P* < 0.05). Serum leptin levels were also significantly decreased by high-molecular-weight SA administration (*P* < 0.05).

**Table 3:** Serum parameters in the experimental mice

	DEN	DEN/High-molecular SA	DEN/Low-molecular SA
AST (IU/l)	163.3 ± 89.3^a^	74.3 ± 31.4^b^	56.9 ± 51.9^b^
ALT (IU/l)	93.6 ± 56.3	46.5 ± 12.8^b^	48.5 ± 24.5
Glucose (mg/dl)	262.6 ± 87.9	242.5 ± 80.0	252.7 ± 54.2
Insulin (μIU/ml)	54.8 ± 34.0	4.3 ± 2.0^b^	7.2 ± 3.4^b^
QUICKI^c^	0.25 ± 0.02	0.34 ± 0.02^b^	0.30 ± 0.02^b^
Adiponectin (ng/ml)	125.2 ± 66.0	129.1 ± 71.7	105.4 ± 67.1
Leptin (pg/ml)	596.5 ± 288.7	277.5 ± 142.9^b^	375.9 ± 202.1
Total cholesterol (mg/dl)	136.4 ± 34.4	130.2 ± 33.5	121.2 ± 21.6
FFA (mEq/L)	0.77 ± 0.28	0.88 ± 0.12	0.64 ± 0.11
Triglyceride (mg/dl)	173.1 ± 67.0	233.4 ± 61.3	225.1 ± 97.8
TNF-α (pg/ml)	64.0 ± 57.1	64.5 ± 41.1	86.4 ± 50.8

a Mean ± SD.

b Significantly different from group 1 by Tukey-Kramer Multiple Comparison Test (P < 0.05).

c QUICKI, quantitative insulin sensitivity check index.

## DISCUSSION

Obesity and related metabolic abnormalities, especially diabetes mellitus and insulin resistance, are critically associated with the development of NAFLD and NASH. These pathophysiological conditions have also been shown to increase the risk of HCC [1–3]. To investigate the preventive effects of SA on the development of obesity-related liver carcinogenesis, MSG-treated mice were selected for the present study because they show significant obesity, hyperinsulinemia, and NASH-like histology, which is virtually undistinguishable from those in humans. Furthermore, the MSG-treated mice were assumed to show high incidence and multiplicity of liver tumors when they were administered DEN [20, 26–28].

Several rodent studies have revealed that intervention using anti-metabolic agents may be an effective strategy for preventing obesity- and NASH-related liver carcinogenesis [10, 11]. In the present study, we investigated the effects of SA for this purpose because it is expected to have beneficial effects on appetite regulation and serum levels of glucose and insulin [15, 29, 30]. Oncogenic effects of insulin, such as stimulation of cell proliferation, on HCC have been reported [31, 32]. In this study, it was considered that suppression of liver tumorigenesis in the obese and diabetic mice was due to, at least in part, reduction of serum insulin levels and improvement in insulin sensitivity. Several animal studies have suggested that targeting higher serum insulin levels and insulin resistance is an effective strategy for inhibiting obesity- and diabetes-related liver tumorigenesis [12, 26]. For instance, dietary supplementation with BCAA, which improves insulin resistance and glucose tolerance in chronic liver disease patients [33], significantly suppresses liver tumorigenesis in obese and diabetic mice by decreasing serum insulin levels as well as improving insulin sensitivity [12]. BCAA supplementation also reduced the risk for HCC in obese patients with liver cirrhosis [3], which demonstrates the clinical significance of lowering insulin levels and attenuating insulin resistance in the prevention of liver carcinogenesis in obese and diabetic patients.

Hepatic steatosis, which is involved in liver tumorigenesis [34], was significantly suppressed by SA administration in the present study. These findings are significant because improvement in steatosis may be a key mechanism of specific agents that inhibit obesity- and NASH-related hepatocarcinogenesis [12, 26, 27, 35]. Recent studies have demonstrated that hepatic *de novo* lipogenesis is increased in NAFLD as a result of overexpression of *SREBP-1c*, which is an important transcription factor that up-regulates genes such as *FAS*, and this promotes fatty acid and TG syntheses [21]. In addition, the hepatic fatty acid oxidative pathway is considered to be a pathophysiological effect in the development of NAFLD [21]. Because the entry flux of fatty acids into mitochondria is regulated by PPAR-α [36, 37], SA attenuated hepatic lipid accumulation and inhibited fatty acid synthesis through the suppression of *FAS* and *SREBP1c* mRNA expression and upregulation of *PPAR-α* mRNA expression in the present study.

In the present study, hepatic lobular inflammation and hepatic mRNA expression levels of inflammatory mediators such as *TNF-α*, *IL-6*, *IL-1β*, *F4/80*, and *CCL2* were decreased in SA-treated mice. SA administration also significantly attenuated chronic inflammation in WAT of the MSG-treated mice. Macrophage infiltration into WAT, which is accompanied by IL-6 and TNF-α production, is an early contributing event for the development of chronic low-grade systemic inflammation [38, 39]. CCL2 plays a crucial role in the recruitment of macrophages into WAT [24, 25] and upregulation of TNF-α, IL-6 and CCL2 in WAT is critically involved in the induction of systemic insulin resistance [38, 39]. Therefore, inhibition of enhanced inflammation in both the liver and adipose tissue by SA supplementation is important in preventing the development of steatosis and subsequent liver tumorigenesis in obese mice. In addition to anti-inflammatory properties, anti-oxidative effects of SA contribute to suppression of obesity-related liver carcinogenesis because hepatic oxidative stress and lipid peroxidation are implicated in the progression of steatosis to NASH and HCC development [22, 23].

Previous studies have explored the importance of alginate viscosity for the anti-metabolic properties. SA is expected to have beneficial effects on postprandial insulinemia because of its high viscosity and gelling properties [30]. Reduction in food intake and suppression of body weight gain after administration of high-viscosity SA, compared to low-viscosity SA, were observed in a previous study [40, 41]. In rats fed a diet supplemented with cholesterol and bile acids, high-viscosity SA administration reduced cholesterol levels, whereas low-viscosity SA did not [41]. In the present study, however, low-molecular-weight SA administration sufficiently attenuated inflammation in both the liver and WAT and suppressed obesity-related liver tumorigenesis. These findings suggest that the low-molecular-weight SA used in this study is sufficient for obtaining these beneficial effects; however, further studies are required to determine the appropriate viscosity of SA, especially considering the clinical use of this agent.

In terms of SA-induced body weight reduction, it can be generally caused by reduced food intake [40, 41]. There is, however, another report indicating that SA does not reduce food consumption [42]. The present study also demonstrates no significant difference was seen in the amount of food intake among groups, but marked reduction of body weights in SA-treated groups. This means reduction of body weight gain in SA-treated groups does not come from reduced food intake, but may come from impaired absorption or increased energy expenditure. The latter seems unfavorable because of more fat tissue in SA-treated groups, although we did not check energy consumption in this study. Although we also did not check and compare muscle mass and the amount of fat and protein in stool, it can be possible that impaired or delayed absorption from intestines induced by SA might cause altered muscle mass and altered balance of fat storage; decreased triglyceride in the liver and increased fat mass in SA-treated groups. In addition, image analyzing by computed tomography [43] could make it possible to compare the mass of muscle, visceral, and subcutaneous fat tissues.

In summary, SA supplementation appears to inhibit the progression of NAFLD and development of liver tumorigenesis. As the previous study indicates that body weight reduction is considered to lower the development of neoplastic lesions [44], the major effect of SA was presumably reducing body weight gain, which led to improving insulin sensitivity, attenuating chronic inflammation, and decreasing oxidative-stress as demonstrated in our study. Supplementation with SA or other weight-reducing agents, therefore, may be an effective strategy for prevention and/or treatment of obesity- and diabetes-related liver tumorigenesis. The results of the present study, together with those of previous studies [12, 26], further strengthen our hypothesis that targeting obesity-induced pathologic conditions, such as insulin resistance, chronic inflammation, and hepatic steatosis, may be effective for preventing liver carcinogenesis in obese individuals [10, 11].

## MATERIALS AND METHODS

### Animals and chemicals

Male MSG-treated ICR mice, which were produced by injecting a dose of MSG (4 mg/g body weight) subcutaneously into newborn ICR mice, were obtained from the Institute for Animal Reproduction (Ibaraki, Japan). The mice were humanely maintained at Gifu University Life Science Research Center in accordance with the Institutional Animal Care Guidelines. Basal diet CRF-1 was purchased from Oriental Yeast (Tokyo, Japan). DEN was purchased from Sigma (St. Louis, MO, USA). Both high- (MW: 1,300,000) and low-molecular-weight (MW: 50,000) SA were kindly supplied by Kaigen Pharmaceutical (Osaka, Japan). The concentration of SA used was 5% (w/w), and this was determined on the basis of previous studies [40, 45]. In clinical practice, 5% SA is used as an approved medication for the treatment of digestive tract damaging without serious side effects.

### Experimental procedure and histopathological examination

The experimental protocol was approved by the Institutional Committee of Animal Experiments of Gifu University. At 2 weeks of age, male MSG-treated ICR mice received a single intraperitoneal injection of DEN (100 mg/kg body weight), and the mice were randomly allocated to 3 groups at 5 weeks of age. The mice in group 1 (n = 12) were provided with a basal diet until the end of the experiment, while the mice in group 2 (n = 12) and group 3 (n = 12) were provided with a basal diet supplemented with 5% high- and low-molecular-weight SA, respectively, until the end of the experiment. At 21 weeks of age, all mice were sacrificed by CO_2_ asphyxiation to investigate the development of NASH, hepatic neoplastic lesions (HCC and liver cell adenoma) and hepatic pre-malignant lesions (foci of cellular alteration: FCA) [46].

Maximum sagittal sections of 3 sublobes (left lateral lobe, left medial lobe, and right medial lobe) were used for histopathological examination. For all experimental groups, 4 μm-thick sections of formalin-fixed and paraffin-embedded livers were stained with hematoxylin & eosin (H&E) for conventional histopathology or with Oil-red O stain to observe liver steatosis. Histological features of the livers were evaluated using the NAFLD activity score (NAS) system [47].

### RNA extraction and quantitative real-time reverse transcription-polymerase chain reaction analysis

Total RNA was isolated from the livers and adipose tissues of the mice usingthe RNeasy Mini Kit and RNeasy Lipid Tissue Mini Kit (Qiagen, Hilden, Germany), respectively. cDNA was amplified from 0.2 mg of total RNA by using the SuperScript III First-Strand Synthesis System (Invitrogen, Carlsbad, CA, USA). Quantitative real-time reverse transcription-PCR (RT-PCR) analysis was performed using specific primers that amplify TNF-α, IL-1β, IL-6, F4/80, chemokine (C-C motif) ligand (CCL)2, fatty acid synthase (FAS), sterol regulatory element-binding protein 1c (SREBP1c), peroxisome proliferator-activated receptor (PPAR)-α, glutathione peroxidase 1 (GPx1), catalase and glyceraldehyde-3-phosphate dehydrogenase (GAPDH) genes. Sequences of these primers have been provided in Table S1. Each sample was analyzed using the LightCycler Nano (Roche Diagnostics, GmbH, Mannheim, Germany) with FastStart Essential DNA Green Master (Roche Diagnostics). Parallel amplification of GAPDH was used as the internal control.

### Clinical chemistry

Blood samples, which were collected from the inferior vena cava of the mice at the time of sacrifice after 8 h of fasting, were used for chemical analyses. Serum levels of TNF-α (R&D Systems, Minneapolis, MN, USA), insulin (Shibayagi, Gunma, Japan), glucose (BioVision Research Products, Mountain View, CA, USA), adiponectin (Shibayagi), leptin (Shibayagi), total cholesterol (Wako Pure Chemical, Osaka, Japan), triglyceride (TG) (Wako Pure Chemical), and free fatty acid (FFA) (Wako Pure Chemical) were determined using enzyme immunoassays, according to the manufacturers' protocols. Serum levels of aspartate aminotransferase (AST) and alanine aminotransferase (ALT) were measured using a standard clinical automatic analyzer (type 7180; Hitachi, Tokyo, Japan). Insulin resistance was estimated by determining the quantitative insulin sensitivity check index (QUICKI) [48].

### Hepatic lipid analysis

After total lipids were extracted from the frozen livers (approximately 100 mg), TG levels were measured using the triglyceride E-test kit (Wako Pure Chemical) [13].

### Oxidative stress analysis

Serum hydroperoxide levels, one of the markers for oxidative stress, were determined using the derivatives of reactive oxygen metabolites (d-ROM) test (FREE Carpe Diem; Diacron s.r.l., Grosseto, Italy) [49].

### Statistical analysis

All data, which are presented as mean ± SD, were analyzed using JMP 11.0 (SAS Institute Inc., Cary, NC, USA). One-way analysis of variance (ANOVA) was used to make comparison between the groups. If ANOVA indicated significant differences, the Tukey-Kramer multiple comparisons test was performed to compare mean values among the groups. Differences were considered significant when the two-sided *P* value was less than 0.05.

## SUPPLEMENTARY TABLE


