# Supplementary material for: Sodium alginate prevents progression of non-alcoholic steatohepatitis and liver carcinogenesis in obese and diabetic mice
Source: Oncotarget. 2016 Feb 8;7(9):10448–58. doi: 10.18632/oncotarget.7249 (PMC4891131; doi:10.18632/oncotarget.7249)
Supplement: Supplementary file 1 [file oncotarget-07-10448-s001.pdf]

SUPPLEMENTARY TABLE

Supplementary Table 1: Primer sequences

Gene	Primer sequences (5'-3')	
	forward	reverse
Catalase	CCTCCTCGTTCAGGATGTGGTT	CGAGGGTCACGAACTGTGTCAG
CCL2	ACTGAAGCCAGCTCTCTCTTCCTC	TTCCTTCTTGGGGTCAGCACAGAC
F4/80	ACAAGACTGACAACCAGACGG	TAGCATCCAGAAGAAGCAGGCGA
FAS	TGGAAAGATAACTGGGTGAC	TGCTGTCGTCTGTAGTCTTG
GAPDH	GACATCAAGAAGGTGGTGAAGCAG	ATACCAGGAAATGAGCTTGACAAA
GPx1	TTTCCCGTGCAATCAGTTC	TCGGACGTACTTGAGGGAAT
IL-1β	CAAGCAACGACAAAATACCTGTG	AGACAAACCGTTTTTCCATCTTCT
IL-6	CCGGAGAGGAGACTTCACAGAG	CTGCAAGTGCATCATCGTTGTT
PPAR-α	AGAGCCCCATCTGTCCTCTC	ACTGGTAGTCTGCAAAACCAAA
SREBP1c	CGGAAGCTGTCGGGGTAG	GTTGTTGATGAGCTGGAGCA
TNF-α	TGGCCCAGACCCTCACACTCAG	ACCCATCGGCTGGCACCACT
